# Improving Prediction Accuracy Using Multi-allelic Haplotype Prediction and Training Population Optimization in Wheat

**DOI:** 10.1534/g3.120.401165

**Published:** 2020-05-05

**Authors:** Ahmad H. Sallam, Emily Conley, Dzianis Prakapenka, Yang Da, James A. Anderson

**Affiliations:** * Department of Plant Pathology,; ^†^ Department of Animal Science, and; ^‡^ Department of Agronomy and Plant Genetics, University of Minnesota, St. Paul, MN 55108

**Keywords:** genomic selection, wheat, plant breeding, training population optimization, haplotype prediction, quantitative trait loci, GenPred, Shared data resources

## Abstract

The use of haplotypes may improve the accuracy of genomic prediction over single SNPs because haplotypes can better capture linkage disequilibrium and genomic similarity in different lines and may capture local high-order allelic interactions. Additionally, prediction accuracy could be improved by portraying population structure in the calibration set. A set of 383 advanced lines and cultivars that represent the diversity of the University of Minnesota wheat breeding program was phenotyped for yield, test weight, and protein content and genotyped using the Illumina 90K SNP Assay. Population structure was confirmed using single SNPs. Haplotype blocks of 5, 10, 15, and 20 adjacent markers were constructed for all chromosomes. A multi-allelic haplotype prediction algorithm was implemented and compared with single SNPs using both *k*-fold cross validation and stratified sampling optimization. After confirming population structure, the stratified sampling improved the predictive ability compared with *k*-fold cross validation for yield and protein content, but reduced the predictive ability for test weight. In all cases, haplotype predictions outperformed single SNPs. Haplotypes of 15 adjacent markers showed the best improvement in accuracy for all traits; however, this was more pronounced in yield and protein content. The combined use of haplotypes of 15 adjacent markers and training population optimization significantly improved the predictive ability for yield and protein content by 14.3 (four percentage points) and 16.8% (seven percentage points), respectively, compared with using single SNPs and *k*-fold cross validation. These results emphasize the effectiveness of using haplotypes in genomic selection to increase genetic gain in self-fertilized crops.

Genomic selection is an important breeding approach for improving quantitative traits. It was advocated as a marker-assisted selection approach that uses high density SNP genotypes for estimating genomic breeding values (Meuwissen *et al.* 2001). Genomic selection relies on linkage disequilibrium (LD) between SNP markers and quantitative trait loci (QTL), where the LD among markers is used as a verification for the association between markers and QTL. Several genomic prediction models were proposed including RR-BLUP, Bayes A, Bayes B, Bayes Cπ, Bayes LASSO, and Reproducing Kernel Hilbert Space RKHS (Meuwissen *et al.* 2001; de Los Campos *et al.* 2009; Kizilkaya *et al.* 2010; Lorenz *et al.* 2011). These prediction methods vary in the assumed genetic effects or/and variance associated with markers. Factors affecting the accuracy of genomic prediction include trait architecture, marker density and LD, training population size, and population structure (Daetwyler *et al.* 2010; Asoro *et al.* 2011; Heffner *et al.* 2011; Lorenz *et al.* 2011; Lorenz *et al.* 2012; Sallam *et al.* 2015; Zhang *et al.* 2016).

Since the development of genomic selection, it has been applied in both animal (Garrick 2011; Rexroad *et al.* 2019) and plant breeding programs (Lian *et al.*, 2014; Sallam *et al.*, 2015; Crossa *et al.*, 2017) resulting in reshaping the breeding approaches by omitting the step of phenotyping the whole population. Rather, a smaller set of a calibration population is phenotyped and genotyped to train a prediction model for estimating breeding values of the selection candidates. The composition of the calibration population is of paramount importance because it determines the efficiency of selecting the best performing individuals. Several studies investigated methods to construct a calibration population for improving the accuracy of genomic prediction including stratified sampling, CDmean optimization, prediction error variance (PEV), and Gmean (Rincent *et al.* 2012; Akdemir *et al.* 2015; Isidro *et al.* 2015; Lorenz and Smith 2015). These methods varied in their improvement of prediction accuracy for traits with different genetic architectures (Isidro *et al.* 2015; Tiede and Smith 2018). One of the important factors determining prediction accuracy is population structure, which can result in variability in allele frequencies and the degree of the genetic relationship between subpopulations/clusters, leading to changes in the accuracy of prediction. The effect of population structure on the accuracy of genomic prediction was observed in both animals (Hayes *et al.* 2009b; Saatchi *et al.* 2011) and plants (Asoro *et al.* 2011; Technow *et al.* 2013), resulting in a general recommendation of constructing a mixed calibration population that includes individuals from all clusters for improving the accuracy of prediction. To cope with structured populations that are developed from parents with different breeding histories, the stratified sampling approach was proposed by sampling a representative sample from each cluster and this approach showed improvement in the prediction accuracy for several quantitative traits (Isidro *et al.* 2015). Unlike PEV and CDmean optimization, the stratified sampling approach is not dependent on trait heritability; thereby, it is expected to perform more consistently across different traits with variable genetic architecture (Rincent *et al.* 2012).

Current methods of genomic selection mostly use single SNP markers to predict the genetic merits of individuals. However, haplotypes may have several advantages over single markers for genomic selection. Phased marker haplotypes can better estimate identity-by-descent and haplotype effects (Meuwissen and Goddard 2000; Hess *et al.* 2017). Additionally, haplotypes increase the LD between the group of phased markers and QTL, explaining greater levels of QTL variance (Hayes *et al.* 2007).

The goal of using genomic selection in plant breeding is to improve the rate of genetic gain under conditions of reduced resources available for evaluating the calibration population. We tested the effect of population structure and using haplotypes on the accuracy of genomic prediction in a wheat population that represents the genetic diversity of the University of Minnesota spring wheat breeding program. The goals of this study were to (1) investigate the effect of population structure on prediction accuracy for yield, test weight, and protein content in a hard red spring wheat population, (2) compare stratified sampling optimization with *k*-fold cross validation for the prediction of the three traits, and (3) compare the prediction accuracy of single markers to four different multi-allelic haplotype blocks with different sizes.

## Materials and Methods

### Phenotypic data

The Minnesota wheat genomic selection (MN-WGS) panel is composed of 383 breeding lines that represent the genetic diversity of the University of Minnesota spring wheat breeding program and includes 93 parents and 290 derived lines from 177 unique crosses represented in their pedigrees (Conley *et al.* 2015). Parents included lines from the spring wheat breeding programs of the University of Minnesota, North Dakota State University, South Dakota State University, AgriPro, WestBred, and CIMMYT. The MN-WGS panel was evaluated together for agronomic traits in two trials in 2013 at St. Paul and Crookston, MN using standard agronomic practices. Plot sizes were 2.6 square meters in St. Paul and 3.4 square meters in Crookston. No fungicides were applied in either location. Lines were planted in a Type II modified augmented field design with 32 blocks. Linkert (Anderson *et al.* 2018) was used as the primary check with LCS Albany (PI 658002), Briggs (Devkota *et al.* 2007), Prosper (Mergoum *et al.* 2013), and Vantage (PI 653518) as secondary checks. Linkert was repeated once in all of the 32 blocks. The population was phenotyped for grain yield, test weight, and protein content. Yield was determined after harvesting plots with a Wintersteiger small plot combine then weighing the grain to express data as kg/ha. The test weight was measured as the weight of seeds that completely fill a quarter pint (118.3 Milliliter) and the resulting data were expressed as kg/hL. Near infrared reflectance spectroscopy (NIR) was used to determine protein content in the harvested grains (Inframatic 9500, Perten Instruments, Sweden).

### Phenotypic data analysis

Correction for spatial field variability for yield, test weight, and protein content was done using a moving grid adjustment (Technow 2015; R-package mvngGrAd, R development core team 2017). After setting the field in rows and columns, a moving mean was calculated using a surrounding grid of a particular size. This moving mean was used subsequently as a covariate to calculate the adjusted phenotypes. A moving average window of eight plots was used to determine the phenotypic performance of the line in the center. For all traits, the entire set of lines were used to correct for variance in trial means using the MIXED procedure in SAS 9.4 (Sallam *et al.* 2015; SAS Institute 2013). In all experiments, genetic and residual variances were calculated using the MIXED procedure in SAS. Broad-sense heritability was estimated using the equation $\text{H}=\sigma_{\text{g}}^{2}/\left(\sigma_{\text{g}}^{2}+\sigma_{\text{e}}^{2}/\text{n}\right)$, where $\sigma_{\text{g}}^{2}$ is genetic variance, $\sigma_{\text{e}}^{2}$ is the variance of random residuals, and n is the number of trials.

### Genotyping and linkage disequilibrium

Leaf tissues were harvested from the 383 breeding lines at the three leaf stage. DNA extraction was performed using the BioSprint 96 DNA Plant Kit according to the manufacturer’s instructions (Qiagen 2016). The panel was genotyped using the 90K Illumina Infinium iSelect Assay. Clustering was performed using Illumina’s Genome Studio Polyploid Clustering Module v1.0 using the procedure described by Wang *et al.* (2014a), followed by manual curation to correct inaccurately clustered loci. Markers were filtered for MAF < 0.05 and missing data > 0.10 resulting in 16,697 SNP markers. From this marker set 14,086 SNP markers had map positions based on a consensus wheat map developed from six independent double haploid mapping populations (Wang *et al.* 2014a). Missing marker data were imputed using LD-kNNi, which imputes missing marker genotypes based on the *k*-nearest neighbor imputation method (Money *et al.* 2015).

To characterize the level of LD in the MN-WGS panel, the adjacent marker LD was estimated as *r^2^* for the 21 wheat chromosomes in TASSEL (Bradbury *et al.* 2007). The genomic additive relationship matrix was estimated among all lines in rrBLUP package of R using all markers (Endelman and Jannink 2012). The genomic additive relationship matrix was estimated as:

$$A=\frac{ZZ^{’}}{2\sum p_{i}(1-p_{i})}$$

where: *Z* = *M* – *P*, *M* being the individuals by SNP loci marker matrix and *P* the frequencies of alleles expressed as 2(*p_i_* – 0.5) with *p_i_* representing the allele frequency of marker *i* (VanRaden 2008).

### Constructing haplotype blocks

The high density SNP marker genotypes were used to construct haplotype blocks after ordering markers based on the consensus map positions for all 21 chromosomes (Wang *et al.* 2014a). We generated four different haplotype blocks, each with a fixed number of 5 adjacent markers (Haploblock-5), 10 (Haploblock-10), 15 (Haploblock-15), and 20 (Haploblock-20) for each chromosome. Haplotype alleles for each haplotype block were numbered using a custom script in R (R Development Core Team 2017).

### Assessment of population structure and training population optimization

A cluster analysis was performed by generating a pairwise distance matrix estimated as 1 - *IBS* (identity-by-state) probability in TASSEL using SNP marker data for all lines in the MN-WGS panel. Using the distance matrix, K-means clustering was performed using the Hartigan-Wong algorithm implemented in R (R Development Core Team 2017). Based on prior knowledge of pedigree information, three clusters were assumed in K-means clustering. Principal component analysis (PCA) was performed in R using SNP marker data for all lines in the MN-WGS panel to visually identify clusters assigned by the K-means clustering (R Development Core Team 2017). Using the genomic additive relationship matrix, the average genetic relationships were calculated for lines within a cluster (*A_ij withing_*) and lines between clusters (*A_ij between_*). To investigate the effect of population structure on genomic prediction in the MN-WGS panel, using single SNP markers only, the three clusters identified by K-means clustering were used in evaluating the predictive ability by combining two clusters for predicting the performance of the third cluster and repeating this step iteratively for all clusters. The predictive ability was calculated as the correlation between phenotypic values of individuals in the validation population and the estimated genomic predictions of those individuals (Legarra *et al.* 2008).

To evaluate genomic prediction accuracy, *k*-fold cross validation was implemented so each individual appeared once in the validation population. We used both single SNP markers and haplotype blocks for the assessment of the predictive ability. The population was randomly shuffled followed by using *k*-fold cross validation by dividing the MN-WGS panel into four groups. One of those groups were excluded to estimate marker/haplotype effects using the three remaining groups to define 75% (288 individuals) of the population as a random calibration population. The *k*-fold cross validation was repeated four times iteratively for each of the four randomly assigned groups. These previous *k*-fold cross validation steps were replicated four times. In addition to *k*-fold cross validation, a training population optimization procedure using stratified sampling was evaluated. For the stratified sampling procedure, clusters identified by K-means clustering were used as a criterion for selecting the calibration population. A stratified sampling genomic prediction procedure was performed by constructing a calibration population through randomly sampling 75% of lines from each of the three clusters. Therefore, the sample size from each cluster was proportional to the size of the cluster, and a total of 75% (288) of lines in the MN-WGS panel were used as a calibration set to predict the remaining 25% (94). The stratified sampling prediction approach was replicated sixteen times using both single SNP markers and the four haplotype block sizes. Each predictive ability value was transformed using Fisher Z. The test statistics were calculated as $T=r\sqrt{n-2}/\sqrt{1-r^{2}}$, where *r* is the predictive ability and *n* is the number of tests (Bobko 2001). The test statistic follows a *t*_N - 2_ distribution (Bobko 2001). A paired *t*-test was used for the assessment of statistical significance between single markers and each haplotype block size for the same constructed calibration populations.

### Genomic prediction models

For genomic best linear unbiased prediction (GBLUP) using single markers, the mixed model with SNP additive effects (or average effects of gene substitution) based on the partition of genotypic values (Da *et al.* 2014) was used:

$$y=1\mu +W_{\alpha }\alpha +e=1\mu +a+e$$

where µ = population mean, **1** = n×1 column vector of 1’s, n = number of lines, **α** = m×1 column vector of marker additive effects, m = number of SNPs, $W_{\alpha }$ = n×m model matrix of **α** with elements of ${\text{2p}}_{2}$, ${\text{p}}_{2}-{\text{p}}_{1}$, and $-{\text{2p}}_{1}$for a marker genotype, $p_{k}$ = frequency of allele k of a SNP (k = 1,2), and $a=W_{\alpha }\alpha$ = GBLUP of additive values of the n lines. Assumptions for the first and second moments are: E(y)=1μ, $\text{Var}(\alpha )=I_{\text{m}}\sigma_{\alpha }^{2}$, and $\text{Var}(e)=R=I_{\text{N}}\sigma_{\text{e}}^{2}$, where $\sigma_{\alpha }^{2}$= variance of SNP additive effects, $\sigma_{\text{e}}^{2}$= residual variance, $I_{\text{m}}$ = m×m identity matrix, and $I_{\text{N}}$= N×N identity matrix. The GBLUP of additive values, and genomic restricted maximum likelihood (GREML) estimates were calculated using the GVCBLUP computer package (Wang *et al.* 2014b; https://animalgene.umn.edu).

For haplotype analysis, a multi-allelic haplotype model that treats each haplotype block as a ‘locus’ and each haplotype within the haplotype block as an allele (Da 2015) was used. The multi-allelic haplotype prediction was modeled as:

$$y=1\mu +W_{\alpha \text{h}}\alpha_{\text{h}}+e=1\mu +a+e$$

where µ = population mean, **1** = n×1 column vector of 1’s, n = number of lines, $\alpha_{h}$ = ${\text{n}}_{\alpha }\times 1$ column vector of haplotype additive effects, ${\text{n}}_{\alpha }$ = number haplotype additive effects (or average effects of gene substitution), $W_{\alpha \text{h}}$ = $n\times {\text{n}}_{\alpha }$ model matrix of $\alpha_{h}$ with elements of ${\text{2p}}_{\text{k}}$, $-(\text{1}-{\text{2p}}_{\text{k}}\text{)}$, and $-2(1-{\text{p}}_{\text{k}}\text{)}$for a haplotype genotype, ${\text{p}}_{\text{k}}$ = frequency of a haplotype in a haplotype block, and $a=W_{\alpha \text{h}}\alpha_{\text{h}}$ = GBLUP of additive values of the n lines. Assumptions for the first and second moments are: E(y)=1μ, $Var(\alpha_{\text{h}})=I_{n\alpha }\sigma_{\alpha h}^{2}$, and $Var(e)=R=I_{N}\sigma_{e}^{2}$, where $\sigma_{\alpha h}^{2}$= variance of haplotype additive effects, $\sigma_{e}^{2}$= residual variance, $I_{n\alpha }$ = ${\text{n}}_{\alpha }\times {\text{n}}_{\alpha }$ identity matrix, and $I_{N}$= N×N identity matrix. The GBLUP of additive values were calculated using the GVCHAP computer package (Prakapenka *et al.* 2020; https://animalgene.umn.edu).

### Data availability

Genotypic and raw phenotypic data for this study are available at figshare portal. The link to the genotypic data (https://figshare.com/articles/Conley_MNWGSpanel_cM_hmp_txt/10031867). The link for the raw phenotypic data (https://figshare.com/articles/Pheno_MN-WGS/10032326). Supplementary tables are available at figshare (https://figshare.com/articles/Supplemental_Tables_for_MN-WGS_panel/10031891). Table S1 includes the average adjacent marker LD estimated as (*r^2^*) for the 21 wheat chromosomes in the MN-WGS panel. Table S2 includes number of haplotype blocks for each chromosome, distance covered by haplotype blocks, maximum number, and average number of haplotype alleles in fixed length haplotypes of 5, 10, 15, and 20 adjacent markers. Table S3 includes the predictive ability for yield, test weight, and protein content using single markers, haplotype blocks of 5 adjacent markers (Haploblock-5), haplotype blocks of 10 adjacent markers (Haploblock-10), haplotype blocks of 15 adjacent markers (Haploblock-15), and haplotype blocks of 20 adjacent markers (Haploblock-20).

## Results

### Phenotypic and genotypic data analysis

**Figure 1:**
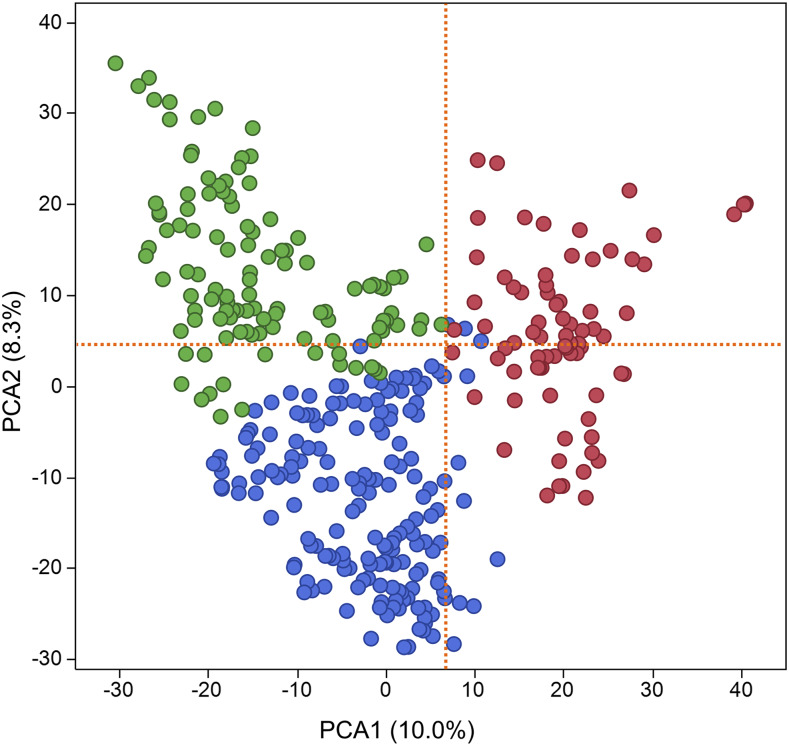
Population stratification of the Minnesota wheat genomic selection (MN-WGS) panel of 383 wheat lines inferred from K-means clustering in which three clusters were identified and visualized on principal component analysis. Cluster 1 is shown in blue, cluster 2 in red, and cluster 3 in green.

**Figure 2:**
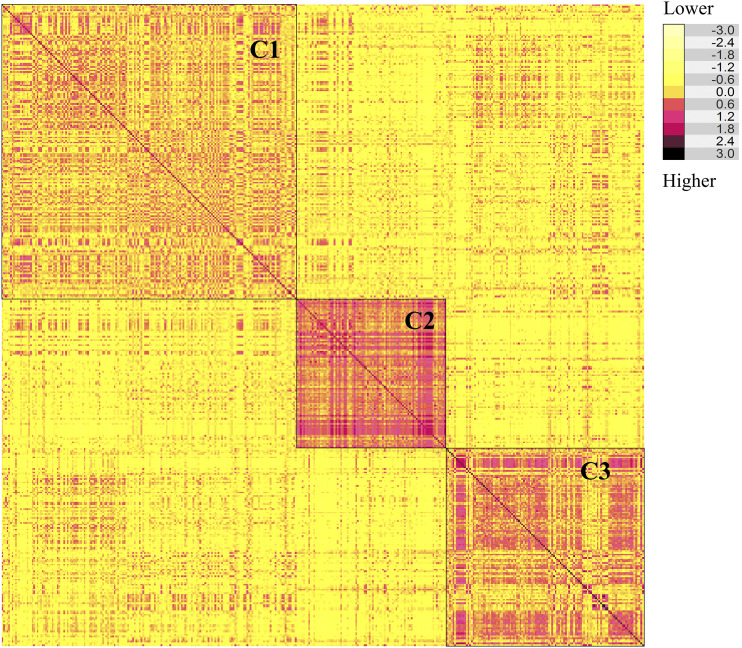
Heatmap for the additive genetic relationship matrix displaying genetic relatedness among lines in the MN-WGS panel with the corresponding clusters identified using K-means clustering.

The MN-WGS panel was evaluated in two balanced trials in Minnesota for grain yield, test weight, and grain protein content. Correction for spatial field variability and trial effects was performed to improve estimates of phenotypic values of individuals. Significant differences were observed among lines for yield, test weight, and protein content. Estimated genetic variance, residual variance, and the broad-sense heritability for each trait are shown in Table 1. Heritability estimates were 0.28 for yield, 0.67 for test weight, and 0.68 for protein content (Table 1). After quality control filtering, 14,086 markers with map positions were used in the study. Marker density varied among chromosomes and ranged from 73 for chromosome 4D to 1,488 for chromosome 2B (Table S1). Extensive levels of LD, estimated as *r^2^*, were observed for all chromosomes that varied between 0.45 for chromosome 7A to 0.69 for chromosome 3B (Table S1). The average adjacent marker LD across all chromosomes was 0.57. K-means clustering identified three different clusters and the number of lines for each cluster were 175 for cluster 1, 89 for cluster 2, and 118 for cluster 3 (Table 2). For the PCA, the majority of individuals in the MN-WGS panel were located in their respective clusters identified by K-means clustering (Figure 1). The first principal component (PC1) explained 10.0% of the variability whereas the second principal component (PC2) explained 8.3% of the variability in the MN-WGS panel (Figure 1). The genomic additive relationship matrix agreed with the results of the K-means clustering in identifying three clusters, each including genetically related individuals (Figure 2). Table 2 displays the average additive genetic relationship between individuals in different (*A_ij between_*) clusters and individuals within (*A_ij within_*) clusters. Cluster 1 had the highest *A_ij between_* and lowest *A_ij within_* compared to the other two clusters (Table 2). On the other hand, cluster 2 had the lowest *A_ij between_* and highest *A_ij within_* (Table 2). The average yield for the three clusters were 5556, 5617, and 5583 kg/ha for cluster 1, cluster 2, and cluster 3; respectively. No significant difference was observed for yield across the three clusters. The average test weight for the three clusters were 79.1, 77.8, and 78.7 kg/hL for cluster 1, cluster 2, and cluster 3; respectively. No significant difference was observed for test weight across the three clusters. The average protein content for the three clusters were 14.4, 14.0, and 14.4% for cluster 1, cluster 2, and cluster 3; respectively. No significant difference was observed for protein content across the three clusters.

**Table 1:** Estimated genetic variance ($\sigma_{\text{g}}^{2}$), residual variance ($\sigma_{\text{e}}^{2}$), and broad-sense heritability (H) for yield, test weight, and protein content in the Minnesota wheat genomic selection panel

Trait	$\sigma_{\text{g}}^{2}$	$\sigma_{\text{e}}^{2}$	H
Yield (kg/ha)	33737	168275	0.29
Test weight (kg/hL)	1.20	1.19	0.67
Protein (%)	0.28	0.27	0.68

**Table 2:** Genetic relationship between (*A_ij between_*) and within (*A_ij within_*) clusters and average predictive ability, when using the cluster in two training populations to predict another cluster, for yield, test weight, and protein based on single markers

				Predictive ability			
Clusters	Number of individuals	*A_ij between_*	*A_ij within_*	Yield	Test weight	Protein	Ave. across traits
Cluster 1	176	−0.12 ± 0.001	0.13 ± 0.002	0.32	0.38	0.28	0.33
Cluster 2	89	−0.16 ± 0.001	0.51 ± 0.005	0.29	0.34	0.19	0.27
Cluster 3	118	−0.14 ± 0.001	0.28 ± 0.003	0.28	0.31	0.23	0.27
Average for each trait				0.30	0.34	0.23	

### Haplotype block construction

Haplotype blocks of 5, 10, 15, and 20 adjacent markers were generated for all chromosomes, with variable number of haplotype alleles identified for each haplotype locus. We will refer to haplotype blocks of 5, 10, 15, and 20 as Haploblock-5, Haploblock-10, Haploblock-15, Haploblock-20; respectively. With the increase of haplotype lengths (Haploblock-5, Haploblock-10, Haploblock-15, to Haploblock-20), lower number of haplotype blocks were generated across the genome with higher numbers of haplotype alleles per haplotype blocks (Table S2). For Haploblock-5, a total of 2,810 haplotype blocks were identified across all chromosomes with up to 29 haplotype alleles per haplotype block (Table S2). On average, across the 21 wheat chromosomes, each Haploblock-5 covered 2.2 cM (Table S2). For Haploblock-10, 1,400 haplotype blocks were identified across all chromosomes with up to 105 haplotype alleles per haplotype block (Table S2). On average across the 21 wheat chromosomes, each Haploblock-10 covered 4.7 cM (Table S2). For Haploblock-15, 930 haplotype blocks were identified across all chromosomes with up to 151 haplotype alleles per haplotype block (Table S2). On average across the 21 wheat chromosomes, each Haploblock-15 covered 7.8 cM (Table S2). For Haploblock-20, 691 haplotype blocks were identified across all chromosomes with up to 259 haplotype alleles per haplotype block (Table S2). On average across the 21 wheat chromosomes, each Haploblock-20 covered 9.6 cM (Table S2). The average number of haplotype alleles across all chromosomes were 3, 4, 6, and 10 for Haploblock-5, Haploblock-10, Haploblock-15, and Haploblock-20; respectively (Table S2). The four different haplotype block sizes were used in genomic prediction using both *k*-fold cross validation and stratified sampling optimization.

### Training population scenarios and comparing between single and haplotype prediction

Generally, the predictive ability was lower for yield compared to test weight and protein content in both *k*-fold cross validation and stratified sampling. To investigate the effect of population structure on the predictive ability, using single SNP markers, the identified clusters were used as training populations by combining two clusters for predicting the third cluster for yield, test weight, and protein content. The size of the formed training populations varied depending on the clusters size (Table 2). The predictive abilities when including a cluster in the training populations are presented in Table 2. When including cluster 1 in the training population in two cases (cluster 1 + cluster 2 and cluster 1 + cluster 3), the predictive abilities were higher across all traits (Table 2). The average predictive abilities for cluster 2 were similar to cluster 3 across all traits and both were lower than cluster 1 (Table 2).

**Figure 3:**
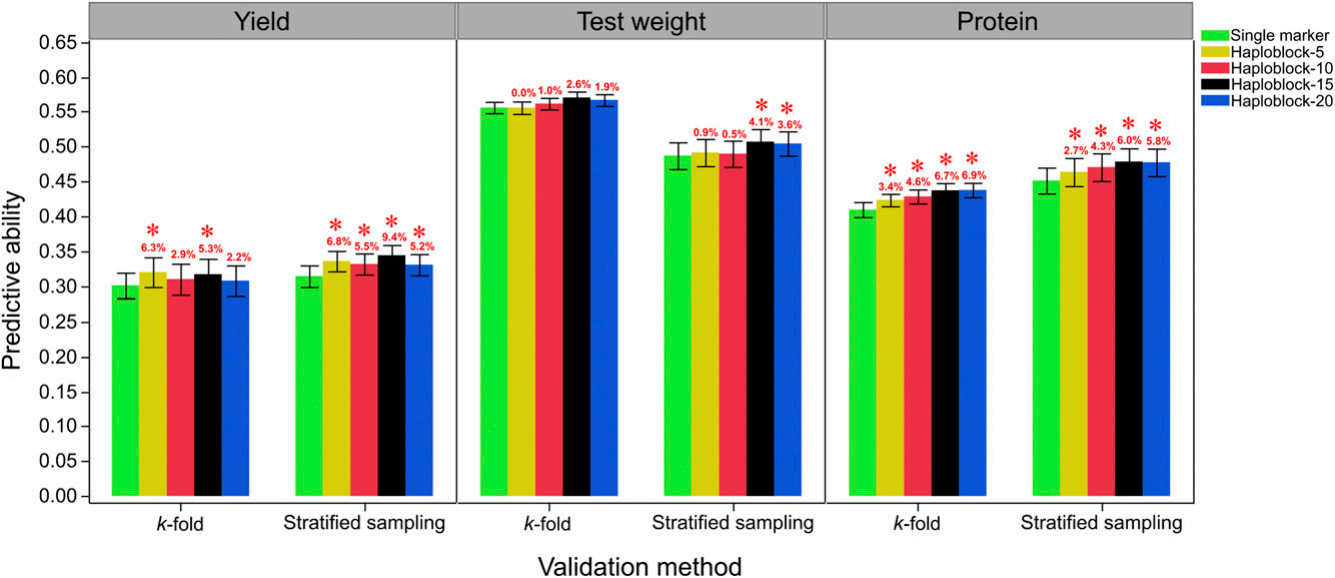
The predictive ability for yield, test weight, and protein content using single markers, haplotype blocks of 5 adjacent markers (Haploblock-5), haplotype blocks of 10 adjacent markers (Haploblock-10), haplotype blocks of 15 adjacent markers (Haploblock-15), and haplotype blocks of 20 adjacent markers (Haploblock-20). The two validation methods used are *k*-fold cross validation and the stratified sampling optimization. A star over the error bar indicates a significant difference in the predictive ability between the haplotype and single markers for the same validation method.

After confirming the effect of population structure on the predictive ability, a training population optimization method was used to design a calibration population by sampling a representative sample from each cluster. In general, the stratified sampling resulted in an increase in the predictive ability compared with *k*-fold cross validation for yield and protein content; while decreasing the predictive ability for test weight using all marker prediction scenarios (Figure 3; Table S3). Four different haplotype block sizes (Haploblock-5, Haploblock-10, Haploblock-15, and Haploblock-20) were used to assess the effectiveness of haplotypes compared with single markers in genomic prediction. All four haplotype blocks improved the predictive ability in both *k*-fold cross validation and stratified sampling in yield and protein content compared with single markers (Figure 3; Table S3). For *k*-fold cross validation in yield, Haploblock-5, Haploblock-10, Haploblock-15, and Haploblock-20 resulted in average increases of 6.3, 2.9, 5.3, and 2.2% in the predictive ability over single markers (Figure 3; Table S3). With the use of stratified sampling, Haploblock-5, Haploblock-10, Haploblock-15, and Haploblock-20 resulted in significant average increases of 6.8, 5.5, 9.4, and 5.2% in the predictive ability over single markers (Figure 3; Table S3). For *k*-fold cross validation in protein content, Haploblock-5, Haploblock-10, Haploblock-15, and Haploblock-20 resulted in significant average increases of 3.4, 4.6, 6.7 and 6.9% in the predictive ability over single markers (Figure 3; Table S3). With the use of stratified sampling, Haploblock-5, Haploblock-10, Haploblock-15, and Haploblock-20 resulted in significant average increases of 2.7, 4.3, 6.0, and 5.8% in the predictive ability for protein content over single markers (Figure 3; Table S3). For test weight, the increase of predictive ability of haplotypes compared with single marker prediction was significant only when using Haploblock-15 and Haploblock-20 with stratified sampling optimization (Figure 3; Table S3).

## Discussion

Most genomic selection investigations rely on using single markers for predicting breeding values of individuals. In our breeding experiment, multi-allelic haplotype prediction performed better than single markers for all three traits investigated. For both single markers and haplotype predictions, the predictive ability for yield was lower than protein content and test weight due to a lower heritability estimate for yield compared with other two traits. Traits with low heritability estimates tend to be highly quantitative, controlled by many loci with smaller effects, and have much environmental noise; which can result in lower prediction accuracies (Bernardo and Yu 2007; Daetwyler *et al.* 2010; Sallam *et al.* 2015). Daetwyler *et al.* (2010) demonstrated that increasing heritability will improve the accuracy of the prediction for both GBLUP and Bayes B. In Norwegian dairy cattle, a strong relationship was observed between prediction accuracy and trait heritability (Luan *et al.* 2009). Similar results were observed in wheat and barley as high heritability traits such as heading date, height, and test weight had higher prediction accuracies compared with low heritability traits such as yield (Heffner *et al.* 2011; Sallam *et al.* 2015). With low heritability traits, a larger number of phenotypic records are needed to better estimate marker effects for improving prediction accuracy (Hayes *et al.* 2009a; Luan *et al.* 2009).

K-means clustering identified three clusters with variable sizes. The cluster analysis revealed the pedigree structure in MN-WGS panel. The three clusters had similar performances for the three traits. The five most frequent parents each appeared in pedigrees at least 45 times (data not shown). One of those parents was not part of the MN-WGS panel. For example, Sabin (Anderson *et al.* 2012), assigned in cluster 2, is a parent to 91 individuals, 87 of which are included in cluster 2. RB07 (Anderson *et al.* 2009), assigned in cluster 3, is a parent to 74 individuals, 71 of which are included in cluster 3. MN02072-7, assigned in cluster 1, is a parent to 66 individuals, 56 of which are included in cluster 1. MN01333-A-2 is a parent to 53 individuals, 41 of which are included in cluster 1. Finally, Glenn (Mergoum *et al.* 2006), assigned in cluster 1, is a parent to 45 individuals, 32 of which are included in cluster 1. These results indicate that clustering in the population is determined by pedigree stratification. Cluster 2 had the highest *A_ij within_* and by searching through pedigree information, we found that all lines in this cluster are half-sibs, sharing Sabin as a common parent. The two training populations that included cluster 2 (cluster 1 + cluster 2 and cluster 2 + cluster 3) had an average predictive ability that is lower than cluster 1, whose included three parents: MN02072-7, Blade (PVP no. 200800075), Faller (Mergoum *et al.* 2008), and Glenn (Mergoum *et al.* 2006) and high frequency of their progenies. Blade, Faller, and Glenn are wheat cultivars and used as parents in the MN-WGS panel but with more progeny for those parents in cluster 1 (48 progeny lines). Several direct progenies of these three parents were also included in cluster 2 (16) and cluster 3 (20), which may explain the highest *A_ij between_* for cluster 1. The high genetic relationship of cluster 1 with the other two clusters resulted in a higher prediction accuracy of single marker prediction when including cluster 1 in the training population. These results may not be applicable to other breeding situations with different levels of population structure due to admixture or pedigree stratification (Toosi *et al.* 2010; Asoro *et al.* 2011). Our findings are in agreement with a genomic selection study in angus beef cattle as one of the five clusters identified using K-means clustering showed lower genetic relationship to other clusters, resulting in the smallest prediction accuracy across all traits when including this cluster in the training population (Saatchi *et al.* 2011). It has been proven that increasing the genetic relationship between the training and validation populations will improve the accuracy of genomic prediction (Habier *et al.* 2007; Lorenz *et al.* 2012; Lorenz and Smith 2015).

The size of the training population is another factor that affects the accuracy of genomic prediction. Training populations including cluster 1 had a larger size compared with the other two clusters and that may contribute to the higher prediction accuracy of cluster 1. Despite the fact that training populations including cluster 3 were larger than cluster 2, both resulted in similar prediction accuracies across all traits. In breeding populations, the change of prediction accuracy due to the increase of training population size is dependent on the genetic relationship (Albrecht *et al.* 2014) and breeding history (Sallam *et al.* 2015); therefore, careful selection of the training population is needed for a successful implementation of genomic selection (Lorenz *et al.* 2012; Lorenz and Smith 2015).

When implementing genomic selection in a breeding program, it is important to consider the best genotypes to be included in the calibration population. Additionally, implementing an efficient marker prediction approach is required to maximize the accuracy of genomic prediction. An effective genomic selection strategy in plant breeding programs is able to design a smaller training population for the purpose of generating genotypic and phenotypic data, which can improve resource allocation (Lorenz 2013; Endelman *et al.* 2014). Several methods were proposed to optimize calibration population design including CDmean (Rincent *et al.* 2012), PEV (Akdemir *et al.* 2015), stratified sampling (Isidro *et al.* 2015), and Gmean (Lorenz and Smith 2015). The stratified sampling approach outperformed CDmean optimization in structured populations across several traits with different genetic architectures (Isidro *et al.* 2015). Compared with *k*-fold cross validation, stratified sampling increased the predictive ability in yield by 4.4, 4.9, 7.0, 8.5 and 7.4% for single markers, Haploblock-5, Haploblock-10, Haploblock-15, and Haploblock-20; respectively. For protein content, the stratified sampling increased the predictive ability by 10.2, 9.5, 9.8, 9.4, and 9.0% for single markers, Haploblock-5, Haploblock-10, Haploblock-15, and Haploblock-20; respectively. Stratified sampling reduced the predictive ability for test weight across all the marker/haplotype genotyping scenarios. The reason for the reduction in the test weight predictive ability when implementing stratified sampling is unknown. Similar results were observed in barley when stratified sampling resulted in a reduction of prediction accuracy in yield across selection cycles while increasing the prediction accuracy for *Fusarium* toxin accumulation (Deoxynivalenol) (Tiede and Smith 2018). There is no absolute training population optimization method that could be applied to all traits with variable genetic architecture (Isidro *et al.* 2015; Tiede and Smith 2018). This may be partly due to the correlation between the traits and population structure, which can affect the accuracy of prediction (Sallam *et al.* 2015). However, other optimization methods performed similarly or more consistently in traits with different genetic architecture and in populations with limited population stratification (Isidro *et al.* 2015; Tiede and Smith 2018).

The current study accentuates the improvement of prediction accuracy based on haplotype blocks *vs.* single marker genomic prediction in a self-fertilized crop species. The four different haplotype sizes significantly improved the accuracy of prediction compared with single marker prediction for yield, test weight, and protein content. It is expected for haplotypes to improve the prediction accuracy over single marker prediction due to the increased LD between haplotypes and causal genetic variants, the effectiveness of capturing genetic relationship using haplotype information, and the ability of haplotype blocks to capture short-range epistatic interactions of nearby genetic variants (Clark 2004; Hayes *et al.* 2007; Hess *et al.* 2017; Jiang *et al.* 2018). Haplotypes may better capture the genomic similarity between lines because LD patterns in each block are considered. A relationship was observed between the length of the haplotype and the accuracy of prediction in animal studies using both simulated (Calus *et al.* 2008; Villumsen *et al.* 2009) and empirical data (Hayes *et al.* 2007; Hess *et al.* 2017). The increase of haplotype length is expected to capture LD between markers in blocks with QTL; thereby increasing the accuracy of prediction. However, this may also increase the number of haplotype allelic classes, which may reduce the accuracy of prediction due to smaller sample sizes representing these classes (Villumsen *et al.* 2009; Da 2015; Hess *et al.* 2017). In our study, the longer haplotype blocks in Haploblock-20 resulted in a large increase for the number of haplotype allelic classes, on average, compared with other haplotype sizes, leading to no improvement in accuracy over Haploblock-15. Villumsen *et al.* (2009) found that the ideal haplotype size could be determined based on the LD level and marker density in a population. In the current study, extensive levels of LD were observed in the four haplotype block sizes with average LD of 0.568, 0.569, 0.572, and 0.570 for Haploblock-5, Haploblock-10, Haploblock-15, and Haploblock-20; respectively. The high levels of LD are a consequence of the selfing nature of wheat that results in extension of LD over long distance. In a simulation study in animals, an adjacent marker LD of 0.20 was sufficient for the use genomic prediction (Calus *et al.* 2008). LD is an important component for driving the accuracy of genomic prediction as the prediction accuracy increases at a similar pattern to the increase of LD (Solberg *et al.* 2008). It is clearly evident that the prediction accuracy is reduced at lower LD levels (Solberg *et al.* 2008; Calus *et al.* 2008). To assess the accuracy of genomic prediction in a rice diversity panel using single markers, LD levels between 0.49 and 0.64 resulted in higher accuracies with reductions in the accuracy of prediction at lower LD levels for three different traits (Ben Hassen *et al.* 2018). Thus, monitoring LD level while constructing haplotype blocks is a safe approach to ensure improvement of genomic predictions. This is because partial linkage between QTL and a group of markers may reduce QTL variance explained by haplotypes, thereby lowering the prediction accuracy (Villumsen *et al.* 2009).

### Utility of haplotype prediction in plant breeding

We evaluated the implementation of a multi-allelic haplotype genomic prediction model in wheat to assess the changes of the predictive ability compared with single markers. Several methods were proposed for constructing haplotype blocks including fixed-length haplotypes and variable-length haplotypes that are based on haplotype identity-by-descent (*IBD*) and LD-based haplotypes (Calus *et al.* 2008; Cuyabano *et al.* 2014; Hess *et al.* 2017). In current study, four fixed numbers (5, 10, 15, and 20) of adjacent markers were used to construct four different haplotype block sizes that resulted in improvement over single marker prediction for traits with different genetic architectures. With the implementation of haplotype prediction in conjunction with a training population optimization approach such as stratified sampling, the prediction accuracy improved substantially. Using Haploblock-15 and implementing stratified sampling for training population optimization, the predictive ability was improved significantly by 14.3 (four percentage points) and 16.8% (seven percentage points) for yield and protein content, respectively, compared with single markers and random *k*-fold cross validation. Improvement of prediction accuracy can change the ranking of top performing individuals in the selection candidate population, thereby increasing genetic gain.

## References

[bib1] Akdemir D., Sanchez J. I., and Jannink J.-L., 2015 Optimization of genomic selection training populations with a genetic algorithm. Genet. Sel. Evol. 47: 38 10.1186/s12711-015-0116-6 25943105 PMC4422310

[bib2] Albrecht T., Auinger H. J., Wimmer V., Ogutu J. O., Knaak C., 2014 Genome-based prediction of maize hybrid performance across genetic groups, testers, locations, and years. Theor. Appl. Genet. 127: 1375–1386. 10.1007/s00122-014-2305-z 24723140

[bib3] Anderson J. A., Linkert G. L., Busch R. H., Wiersma J. J., Kolmer J. A., 2009 Registration of ‘RB07’ wheat. J. Plant Regist. 3: 175–180. 10.3198/jpr2008.08.0478crc

[bib4] Anderson J. A., Wiersma J. J., Linkert G. L., Kolmer J. A., Jin Y., 2012 Registration of ‘Sabin’ wheat. J. Plant Regist. 6: 174–179. 10.3198/jpr2011.06.0344crc

[bib5] Anderson J. A., Wiersma J. J., Linkert G. L., Reynolds S., Kolmer J. A., 2018 Registration of ‘Linkert’ spring wheat with good straw strength and adult plant resistance to the Ug99 family of stem rust races. J. Plant Registrations 12: 208–214. 10.3198/jpr2017.07.0046crc

[bib6] Asoro F. G., Newell M. A., Beavis W. D., Scott M. P., and Jannink J.-L., 2011 Accuracy and training population design for genomic selection on quantitative traits in elite North American oats. Plant Genome 4: 132–144. 10.3835/plantgenome2011.02.0007

[bib7] Bernardo R., and Yu J., 2007 Prospects for genome-wide selection for quantitative traits in maize. Crop Sci. 47: 1082–1090. 10.2135/cropsci2006.11.0690

[bib8] Bobko P., 2001 Correlation and regression: Application for industrial organizational psychology and management, Ed. 2nd Sage Publications, Inc., Thousand Oaks, CA, . 10.4135/9781412983815

[bib9] Ben Hassen M., Cao T. V., Bartholomé J., Orasen G., Colombi C., 2018 Rice diversity panel provides accurate genomic predictions for complex traits in the progenies of biparental crosses involving members of the panel. Theor. Appl. Genet. 131: 417–435. 10.1007/s00122-017-3011-4 29138904 PMC5787227

[bib10] Bradbury P. J., Zhang Z., Kroon D. E., Casstevens T. M., Ramdoss Y., 2007 TASSEL: Software for association mapping of complex traits in diverse samples. Bioinformatics 23: 2633–2635. 10.1093/bioinformatics/btm308 17586829

[bib11] Calus M. P. L., Meuwissen T. H. E., De Roos A. P. W., and Veerkamp R. F., 2008 Accuracy of genomic selection using different methods to define haplotypes. Genetics 178: 553–561. 10.1534/genetics.107.080838 18202394 PMC2206101

[bib12] Clark A. G., 2004 The role of haplotypes in candidate gene studies. Genet. Epidemiol. 27: 321–333. 10.1002/gepi.20025 15368617

[bib13] Conley, E. J., L. Gao, and J. A. Anderson, 2015 Exploration of genomic selection strategies to complement wheat FHB resistance breeding. Presented at: National Fusarium Head Blight Forum; 2015 Dec 6–8; St. Louis, MO. http://z.umn.edu/ejc14

[bib14] Crossa J., Pérez-Rodríguez P., Cuevas J., Montesinos-López O., Jarquín D., 2017 Genomic selection in plant breeding: Methods, models, and perspectives. Trends Plant Sci. 22: 961–975. 10.1016/j.tplants.2017.08.011 28965742

[bib15] Cuyabano B. C., Su G., and Lund M. S., 2014 Genomic prediction of genetic merit using LD-based haplotypes in the Nordic Holstein population. BMC Genomics 15: 1171 10.1186/1471-2164-15-1171 25539631 PMC4367958

[bib16] Da Y., 2015 Multi-allelic haplotype model based on genetic partition for genomic prediction and variance component estimation using SNP markers. BMC Genet. 16: 144 10.1186/s12863-015-0301-1 26678438 PMC4683770

[bib17] Da Y., Wang C., Wang S., and Hu G., 2014 Mixed model methods for genomic prediction and variance component estimation of additive and dominance effects using SNP markers. PLoS One 9: e87666 10.1371/journal.pone.0087666 24498162 PMC3907568

[bib18] Daetwyler H. D., Pong-Wong R., Villanueva B., and Woolliams J. A., 2010 The impact of genetic architecture on genome-wide evaluation methods. Genetics 185: 1021–1031. 10.1534/genetics.110.116855 20407128 PMC2907189

[bib19] de Los Campos G., Gianola D., and Rosa G. J., 2009 Reproducing kernel Hilbert spaces regression: a general framework for genetic evaluation. J. Anim. Sci. 87: 1883–1887. 10.2527/jas.2008-1259 19213705

[bib20] Devkota R. N., Rudd J. C., Jin Y., Glover K. D., Hall R. G., 2007 Registration of ‘Briggs’. Wheat. Crop Sci. 47: 432–434. 10.2135/cropsci2006.07.0503

[bib21] Endelman J. B., Atlin G. N., Beyene Y., Semagn K., Zhang X., 2014 Optimal design of preliminary yield trials with genome-wide markers. Crop Sci. 54: 48–59. 10.2135/cropsci2013.03.0154

[bib22] Endelman J. B., and Jannink J.-L., 2012 Shrinkage estimation of the realized relationship matrix. G3 (Bethesda) 2: 1405–1413. 10.1534/g3.112.004259 23173092 PMC3484671

[bib23] Garrick D. J., 2011 The nature, scope and impact of genomic prediction in beef cattle in the United States. Genet. Sel. Evol. 43: 17 10.1186/1297-9686-43-17 21569623 PMC3107171

[bib24] Habier D., Fernando R. L., and Dekkers J. C. M., 2007 The impact of genetic relationship information on genome-assisted breeding values. Genetics 177: 2389–2397. 10.1534/genetics.107.081190 18073436 PMC2219482

[bib25] Hayes B. J., Bowman P. J., Chamberlain A. J., and Goddard M. E., 2009 a Invited review: Genomic selection in dairy cattle: Progress and challenges. J. Dairy Sci. 92: 433–443. 10.3168/jds.2008-1646 19164653

[bib26] Hayes B. J., Bowman P. J., Chamberlain A. C., Verbyla K., and Goddard M. E., 2009 b Accuracy of genomic breeding values in multi-breed dairy cattle populations. Genet. Sel. Evol. 41: 51 10.1186/1297-9686-41-51 19930712 PMC2791750

[bib27] Hayes B. J., Chamberlain A. J., McPartlan H., MacLeod I., Sethuraman L., 2007 Accuracy of marker-assisted selection with single markers and marker haplotypes in cattle. Genet. Res. 89: 215–220. 10.1017/S0016672307008865 18208627

[bib28] Heffner E. L., Jannink J.-L., and Sorrells M. E., 2011 Genomic selection accuracy using multifamily prediction models in a wheat breeding program. Plant Genome 4: 65–75. 10.3835/plantgenome2010.12.0029

[bib29] Hess M., Druet T., Hess A., and Garrick D., 2017 Fixed-length haplotypes can improve genomic prediction accuracy in an admixed dairy cattle population. Genet. Sel. Evol. 49: 54 10.1186/s12711-017-0329-y 28673233 PMC5494768

[bib30] Isidro J., Jannink J.-L., Akdemir D., Poland J., Heslot N., 2015 Training set optimization under population structure in genomic selection. Theor. Appl. Genet. 128: 145–158. 10.1007/s00122-014-2418-4 25367380 PMC4282691

[bib31] Jiang Y., Schmidt R. H., and Reif J. C., 2018 Haplotype-based genome-wide prediction models exploit local epistatic interactions among markers. G3 (Bethesda) 8: 1687–1699. 10.1534/g3.117.300548 29549092 PMC5940160

[bib32] Kizilkaya K., Fernando R. L., and Garrick D. J., 2010 Genomic prediction of simulated multibreed and purebred performance using observed fifty thousand single nucleotide polymorphism genotypes. J. Anim. Sci. 88: 544–551. 10.2527/jas.2009-2064 19820059

[bib33] Legarra A., Robert-Granié C., Manfredi E., and Elsen J. M., 2008 Performance of genomic selection in mice. Genetics 180: 611–618. 10.1534/genetics.108.088575 18757934 PMC2535710

[bib34] Lian L., Jacobson A., Zhong S., and Bernardo R., 2014 Genomewide prediction accuracy within 969 maize biparental populations. Crop Sci. 54: 1514–1522. 10.2135/cropsci2013.12.0856

[bib35] Lorenz A. J., 2013 Resource allocation for maximizing prediction accuracy and genetic gain of genomic selection in plant breeding: A simulation experiment. G3 (Bethesda) 3: 481–491. 10.1534/g3.112.004911 23450123 PMC3583455

[bib36] Lorenz A. J., Chao S., Asoro F. G., Heffner E. L., Hayashi T., 2011 Genomic selection in plant breeding: Knowledge and prospects. Adv. Agron. 110: 77–123. 10.1016/B978-0-12-385531-2.00002-5

[bib37] Lorenz A. J., and Smith K. P., 2015 Adding genetically distant individuals to training populations reduces genomic prediction accuracy in Barley. Crop Sci. 55: 2657–2667. 10.2135/cropsci2014.12.0827

[bib38] Lorenz A. J., Smith K. P., and Jannink J.-L., 2012 Potential and optimization of genomic selection for Fusarium head blight resistance in six-row barley. Crop Sci. 52: 1609–1621. 10.2135/cropsci2011.09.0503

[bib39] Luan T., Woolliams J. A., Lien S., Kent M., Svendsen M., 2009 The accuracy of genomic selection in Norwegian red cattle assessed by cross-validation. Genetics 183: 1119–1126. 10.1534/genetics.109.107391 19704013 PMC2778964

[bib40] Mergoum M., Frohberg R. C., Olson T., Friesen T. L., Rasmussen J. B., 2006 Registration of ‘Glenn’ wheat. Crop Sci. 46: 473–474. 10.2135/cropsci2005.0287

[bib41] Mergoum M., Frohberg R. C., Stack R. W., Rasmussen J. W., and Friesen T. L., 2008 Registration of ‘Faller’ Spring Wheat. J. Plant Regist. 2: 224–229. 10.3198/jpr2008.03.0166crc

[bib42] Mergoum M., Frohberg R. C., Stack R. W., Simsek S., Adhikari T. B., 2013 ‘Prosper’: a high-yielding hard red spring wheat cultivar adapted to the north central plains of the USA. J. Plant Regist. 7: 75–80. 10.3198/jpr2012.05.0271crc

[bib43] Meuwissen T. H. E., and Goddard M. E., 2000 Fine mapping of quantitative trait loci using linkage disequilibria with closely linked marker loci. Genetics 155: 421–430. 10790414 10.1093/genetics/155.1.421 PMC1461086

[bib44] Meuwissen T. H. E., Hayes B. J., and Goddard M. E., 2001 Prediction of total genetic value using genome-wide dense marker maps. Genetics 157: 1819–1829. 11290733 10.1093/genetics/157.4.1819 PMC1461589

[bib45] Money D., Gardner K., Migicovsky Z., Zhong G.-Y., Schwaninger H., 2015 LinkImpute: Fast and accurate genotype imputation for nonmodel organisms. G3 (Bethesda) 5: 2383–2390. 10.1534/g3.115.021667 26377960 PMC4632058

[bib46] Prakapenka D., Wang C., Liang Z., Bian C., Tan C., 2020 GVCHAP: A computing pipeline for genomic prediction and variance component estimation using haplotypes and SNP markers. Front. Genet. 11: 282 10.3389/fgene.2020.00282 32318093 PMC7154123

[bib47] R Development Core Team, 2017 R: A language and environment for statistical computing, R Foundation for Statistical Computing, Vienna, Austria.

[bib48] Rexroad C., Vallet J., Matukumalli L. K., Reecy J., Bickhart D., 2019 Genome to phenome: Improving animal health, production, and well-being - A new USDA blueprint for animal genome research 2018–2027. Front. Genet. 10: 327 10.3389/fgene.2019.00327 31156693 PMC6532451

[bib49] Rincent R., Laloë D., Nicolas S., Altmann T., Brunel D., 2012 Maximizing the reliability of genomic selection by optimizing the calibration set of reference individuals: Comparison of methods in two diverse groups of maize inbreds (*Zea mays* L.). Genetics 192: 715–728. 10.1534/genetics.112.141473 22865733 PMC3454892

[bib50] Saatchi M., McClure M. C., McKay S. D., Rolf M. M., Kim J., 2011 Accuracies of genomic breeding values in American Angus beef cattle using K-means clustering for cross-validation. Genet. Sel. Evol. 43: 40 10.1186/1297-9686-43-40 22122853 PMC3250932

[bib51] Sallam A. H., Endelman J. B., Jannink J.-L., and Smith K. P., 2015 Assessing genomic selection prediction accuracy in a dynamic barley breeding population. Plant Genome 8: 1–15. 10.3835/plantgenome2014.05.0020 33228279

[bib52] SAS Institute Inc, 2013 Base SAS 9.4. Procedures guide: Statistical procedures, SAS Institute Inc., Cary, NC.

[bib53] Solberg T. R., Sonesson A. K., Woolliams J. A., and Meuwissen T. H. E., 2008 Genomic selection using different marker types and densities. J. Anim. Sci. 86: 2447–2454. 10.2527/jas.2007-0010 18407980

[bib54] Technow F., Bürger A., and Melchinger A. E., 2013 Genomic prediction of northern corn leaf blight resistance in maize with combined or separated training sets for heterotic groups. G3 (Bethesda) 3: 197–203. 10.1534/g3.112.004630 23390596 PMC3564980

[bib55] Technow, F., 2015 R package mvngGrAd: moving grid adjust-ment in plant breeding field trials. R package version 0.1.5.

[bib56] Tiede T., and Smith K. P., 2018 Evaluation and retrospective optimization of genomic selection for yield and disease resistance in spring barley. Mol. Breed. 38: 55 10.1007/s11032-018-0820-3

[bib57] Toosi A., Fernando R. L., and Dekkers J. C. M., 2010 Genomic selection in admixed and crossbred populations. J. Anim. Sci. 88: 32–46. 10.2527/jas.2009-1975 19749023

[bib58] VanRaden P. M., 2008 Efficient methods to compute genomic predictions. J. Dairy Sci. 91: 4414–4423. 10.3168/jds.2007-0980 18946147

[bib59] Villumsen T. M., Janss L., and Lund M. S., 2009 The importance of haplotype length and heritability using genomic selection in dairy cattle. J. Anim. Breed. Genet. 126: 3–13. 10.1111/j.1439-0388.2008.00747.x 19207924

[bib60] Wang S., Dolferus R., Appels R., Dubcovsky J., Maccaferri M., 2014a Characterization of polyploid wheat genomic diversity using a high-density 90 000 single nucleotide polymorphism array. Plant Biotechnol. J. 12: 787–796. 10.1111/pbi.12183 24646323 PMC4265271

[bib61] Wang C., Prakapenka D., Wang S., Pulugurta S., Runesha H. B., 2014b GVCBLUP: A computer package for genomic prediction and variance component estimation of additive and dominance effects. BMC Bioinformatics 15: 270 10.1186/1471-2105-15-270 25107495 PMC4133608

[bib62] Zhang X., Sallam A., Gao L., Kantarski T., Poland J., 2016 Establishment and optimization of genomic selection to accelerate the domestication and improvement of intermediate wheatgrass. Plant Genome 9: 1–18. 10.3835/plantgenome2015.07.0059 27898759

